# Prevalence of Loss of All Teeth (Edentulism) and Associated Factors in Older Adults in China, Ghana, India, Mexico, Russia and South Africa †

**DOI:** 10.3390/ijerph111111308

**Published:** 2014-10-30

**Authors:** Karl Peltzer, Sandra Hewlett, Alfred E. Yawson, Paula Moynihan, Raman Preet, Fan Wu, Godfrey Guo, Perianayagam Arokiasamy, James J. Snodgrass, Somnath Chatterji, Mark E. Engelstad, Paul Kowal

**Affiliations:** 1 HIV/AIDS/STIs and TB (HAST), Human Sciences Research Council, Private Bag X41, Pretoria 0001, South Africa; 2 Department of Psychology, University of the Free State, Bloemfontein 9300, South Africa; 3 ASEAN Institute for Health Development, Madidol University, Salaya, Phutthamonthon, Nakhonpathom 73170, Thailand; 4 Department of Restorative Dentistry, University of Ghana Dental School, College of Health Sciences, Korle-Bu, Accra KB 460, Ghana; E-Mail: sandrahewlett@yahoo.co.uk; 5 Department of Community Health, University of Ghana Medical School, College of Health Sciences, Korle-Bu, KB 4236, Accra, Ghana; E-Mail: aeyawson@yahoo.com; 6 Institute of Health & Society, Centre for Oral Health Research, Newcastle University, Newcastle NE2 4BW, UK; E-Mail: paula.moynihan@newcastle.ac.uk; 7 Faculty of Medicine, Department of Public Health and Clinical Medicine, Epidemiology and Global Health, Umeå University, Umeå SE-90187, Sweden; E-Mail: raman.preet@epiph.umu.se; 8 Shanghai municipal Center for Disease Control and Prevention, No. 1380 West Zhongshan Rd, Changning District, Shanghai 200336, China; E-Mails: fwu@scdc.sh.cn (F.W.); guoyanfei@scdc.sh.cn (G.G.); 9 International Institute for Population Studies, Mumbai, Maharashtra 400088, India; E-Mail: parokiasamy@yahoo.co.uk; 10 Department of Anthropology, University of Oregon, Eugene, OR 97403, USA; E-Mail: jjosh@uoregon.edu; 11 World Health Organization, SAGE, 1211 Geneva 27, Switzerland; E-Mails: chatterjis@who.int (S.C.); kowalp@who.int (P.K.); 12 Department of Oral and Maxillofacial Surgery, Oregon Health and Science University, Portland OR 97201, USA; E-Mail: engelsta@ohsu.edu; 13 University of Newcastle Research Centre for Gender, Health and Ageing, Newcastle, NSW 2305, Australia

**Keywords:** edentulism, associated factors, China, Ghana, India, Mexico, Russia, South Africa

## Abstract

Little information exists about the loss of all one’s teeth (edentulism) among older adults in low- and middle-income countries. This study examines the prevalence of edentulism and associated factors among older adults in a cross-sectional study across six such countries. Data from the World Health Organization (WHO’s) Study on global AGEing and adult health (SAGE) Wave 1 was used for this study with adults aged 50-plus from China (*N* = 13,367), Ghana (*N* = 4724), India (*N* = 7150), Mexico (*N* = 2315), Russian Federation (*N* = 3938) and South Africa (*N* = 3840). Multivariate regression was used to assess predictors of edentulism. The overall prevalence of edentulism was 11.7% in the six countries, with India, Mexico, and Russia has higher prevalence rates (16.3%–21.7%) than China, Ghana, and South Africa (3.0%–9.0%). In multivariate logistic analysis sociodemographic factors (older age, lower education), chronic conditions (arthritis, asthma), health risk behaviour (former daily tobacco use, inadequate fruits and vegetable consumption) and other health related variables (functional disability and low social cohesion) were associated with edentulism. The national estimates and identified factors associated with edentulism among older adults across the six countries helps to identify areas for further exploration and targets for intervention.

## 1. Introduction

Edentulism is the state of having lost all of one’s natural teeth [1]. Monitoring the occurrence of an oral “end state” such as edentulism is important because it is an indicator of both population health and the functioning and adequacy of a country's oral health care system [2]. The 2010 Global Burden of Disease Study results shows a steady decline in age-standardized population (Disability Adjusted Life Year) DALY rates for edentulism, from 144/100,000 in 1990 to 89/100,000 [3]. An even more recent review Kossioni [4] revealed that data on the oral health of community-dwelling (living in their own homes, not institutionalised) older people are scarce in many parts of the world, particularly in Africa, Asia and South America, and direct comparisons are not always possible due to methodological variations. Wu *et al.* [5] found a wide variation in edentulism prevalence among adults aged 50 and above in five ethnic groups in the United States: Asians, African Americans, Hispanics, Native Americans, and non-Hispanic Caucasians. In 2008, Native Americans had the highest predicated rate of edentulism based on oral exam (24%), followed by African Americans (19%), Caucasians (17%), Asians (14%), and Hispanics (14%). Dolan *et al.* [6] found that in persons 45 years and older in the USA that a total of 19% of subjects were edentulous by self-report, and Medina-Solís *et al.* [7] found that the prevalence of self-reported edentulism ranged from 2% in the 35–44 year age group to 26% in the 65–74 year age group in Mexico. Among Europeans, Müller *et al.* [8] observed in a review of clinical and cross-sectional interview studies that in the 1990s the prevalence of edentulism among 75-year-olds in a Swedish, Danish and Finnish city were 27%, 45% and 58% respectively. In 2003, Mojon [9] reports a range of 0% to 72% of the 65 to 74 year age group in Europe. The 2009 UK Adult Dental Health Survey self-reported that the percentage of adults who were edentulous was 5% for those aged 55–64 years and 15% for those aged 65–74 years [10].

Factors associated with edentulism include: (1) socioeconomic factors such as increasing age [11,12,13,14,15], being female [12,13,16], lower education [14,17], lower economic status [6,12,16,17,18], lower social class [16], health security [11], and rural residence [19]; (2) chronic conditions such as asthma [20], diabetes [20,21], arthritis [20], angina pectoris [22], stroke [23], hypertension [24] and obesity [25]; (3) health risk behaviour including smoking [12,15,20,26,27,28], former smoking [15,26], inadequate consumption of fruit and vegetables [29,30] and infrequent dental visits [11]; and, (4) other health-related factors, including functional disability [31], lower scores on cognitive testing [32,33], poorer self-rated level of general health [6], social cohesion [16,34], and self-esteem and quality of life [33,35,36]. Steele *et al.* [37] observed that social support is a determinant of oral health-related quality of life, and that psychosocial factors, such as loneliness and social isolation, were associated with onset of periodontal disease [38]. Social networks were associated with better self-rated oral health, and social capital had beneficial effects on the number of teeth [39]. 

The aim of this study was to investigate the prevalence of edentulism and associated factors among older adults in six lower- and upper-middle income countries. The objectives include: (1) to estimate the prevalence of self-reported edentulism in older adults, and (2) to identify possible factors such as sociodemographics, chronic conditions, health risk behaviour and other health related variables associated edentulism in six lower- and upper-middle income countries.

## 2. Methods

### 2.1. Sample and Procedure

Study on global AGEing and adult health (SAGE) is a longitudinal study of ageing, health and wellbeing with nationally representative cohorts of persons aged 50 years and older in China, Ghana, India, Mexico, the Russian Federation and South Africa, along with comparison samples of younger adults aged 18–49 years in each country [40]. Further information about the SAGE survey design and methods is available from a published data resource profile [40]. A broad range of health and health-related determinants and outcomes were measured as part of SAGE, including questions about oral health. SAGE Wave 1 was implemented as a face-to-face household interview between 2007 and 2010. All six countries implemented multistage cluster sampling strategies which resulted in nationally representative cohorts of older adults [41]. SAGE has been approved by the World Health Organization’s Ethical Review Board. Additionally, each partner organization obtained ethical clearance through their respective review bodies. Informed consent has been obtained from all study participants. The study complies with the STROBE statement.

### 2.2. Measures

A standardized survey instrument, including modules on health and its determinants, disability, risk factors, chronic conditions, anthropometric measurements (height, weight, waist and hip circumferences), blood pressure measures and cognition, were used across all six SAGE countries [40]. The procedures for including country-specific adaptations to the standardized questionnaire and translations into local languages from English follow those developed by and used for the World Health Survey [42]. The main outcome, edentulism, was assessed by self-report, “Have you lost all of your natural teeth?” Response options were “yes”and “no”. 

Sociodemographic covariates:

Age, sex, years of education completed, place of residence (urban or rural), and economic status was obtained through self-report. Urban and rural categories were defined by each country based on census definitions. Economic status was estimated through wealth levels generated using a multi-step process, whereby asset ownership was converted to an asset ladder, Bayesian post-estimation method was used to generate raw continuous income estimates and these were then transformed into quintiles [43].

Chronic conditions covariates:

Blood pressure (systolic and diastolic) was measured three times on the right arm/wrist of the seated respondent using an automated recording device (OMRON R6 Wrist Blood Pressure Monitor, HEM-6000-E, Omron Healthcare Europe, B.V., Hoofddorp, and The Netherlands). Out of three measurements, the average of the last two readings was used. In accordance with the Seventh Report of the Joint National Committee on Prevention, Detection, Evaluation, and Treatment of High Blood Pressure, individuals with systolic blood pressure ≥ 140 mmHg and/or diastolic blood pressure ≥ 90 mmHg and/or who reported the current use of antihypertensive medication were considered to be suffering from high blood pressure [44]. A further set of chronic conditions was assessed through self-report, with respondents asked if they had been diagnosed with a number of chronic conditions, including, angina, arthritis, asthma, and diabetes.

Anthropometric Measurements:

Participants were weighed and measured by trained researchers using standardised protocols [40]. Standing height was measured to the nearest 0.1 cm without shoes, using a stadiometer. Participants wearing light clothes, were weighed to the nearest 0.01 kg, on a load-cell-operated digital scale which was first calibrated using a standard weight and re-checked daily [40]. Body mass index (BMI) was calculated as weight in kg divided by height in metres squared. Participants underweight, normal eight, pre-obese and obesity were defined as ≤18.5, 18.5–24.9, ≥25.0–29.9 kg/m^2^ and ≥30 kg/m^2^, respectively [45].

Health risk behaviour covariates:

Tobacco use. The first question asked ‘Have you ever smoked tobacco or used smokeless tobacco?’ Those who responded with “yes” were asked ‘Do you currently use (smoke, sniff or chew) any tobacco products such as cigarettes, cigars, pipes, chewing tobacco or snuff?’ The response options were ‘Yes, daily’, ‘Yes, but not daily’ and ‘No, not at all’. Everybody was asked, “In the past, did you ever smoke tobacco or use smokeless tobacco daily?” These questions are based on the STEPwise approach to surveillance (STEPS) of Non-Communicable Disease (NCD) risk factors [46].

Fruit and vegetable consumption was estimated by 24 h dietary recall using two questions ‘How many servings of fruit do you eat on a typical day?’ and ‘How many servings of vegetables do you eat on a typical day?’. Researchers were trained to show all respondents a nutrition risk factor card that indicates both in writing and pictorially general categories, amounts, and examples of fruit and vegetables in an attempt to standardize the serving size and number of servings reported. The nutrition card categorized one serving of vegetables into one of three groups: (a) one cup of raw green leafy vegetables such as spinach or salad; (b) one cup of other vegetables cooked or chopped raw, such as tomatoes, carrots, pumpkin, corn, Chinese cabbage, beans, or onions; and (c) one-half cup of vegetable juice. Furthermore, the nutrition card categorized one serving of fruit into one of three groups: (a) one medium-sized piece of fruit, such as an apple, banana, or orange; (b) one-half cup of cooked, chopped, or canned fruit; and (c) one-half cup of fruit juiced, not artificially flavored (4). Insufficient fruit and vegetable consumption was defined as less than five servings of fruits and/or vegetables a day [47]. 

Other health related risk covariates:

Functional disability was measured by the 12-item WHO Disability Assessment Schedule, version 2.0 (WHODAS-2) [48]. Participants were asked about difficulties in the last 30 days with performing activity of daily living-type and instrumental activity of daily living-type questions. Responses to these questions were scored using a five-point Likert-type response scale: ‘none’, ‘mild’, ‘moderate’, ‘severe’ and ‘extreme/cannot do’. The computed WHODAS-2 score ranged from 0 to 48 and was later transformed into a score of 0–100 with 100 being severe or extreme disability [48]. WHODAS-2 subscales and summary indices were coded using the International Classification of Functioning, Disability and Health (ICF) disability categories [49], namely: No problem (0%–4%); Mild problem (5%–24%); Moderate problem (25%–49%); Severe problem (50%–95%); Extreme problem (95%–100%); and then dichotomised into ≥25% = 1 and <25% = 0 for analysis. Cronbach’s alpha for the WHODAS-2 was 0.92 in this sample.

Social cohesion was measured with nine items, starting with the introduction ‘How often in the last 12 months have you...’ e.g., attended any group, club, society, union, or organisational meeting?’ Response options ranged from never = 1 to daily = 5. The scores assigned to each of the items were ‘never’ (1), ‘once or twice a year’ (2), ‘once or twice per month’ (3), ‘once or twice per week’ (4), and ‘daily’ (5) were summed. These responses were used to create a single score and variable for overall social cohesion [50]. Cronbach’s alpha for this social cohesion index in this sample was 0.81.

Subjective overall general health status was assessed with one question: “In general, how would you rate your health today?” Response options ranged from 1 = very good to 5 = very bad. This was dichotomised into 1 = very good or good and 0 = moderate to very bad.

Cognitive functioning was assessed with one question, “Overall in the last 30 days, how much difficulty did you have with concentrating or remembering things?” Response options ranged from 1 = none to 5 = extreme or cannot do. Severe or extreme/cannot do is classified as poor cognitive functioning.

### 2.3. Data Analysis

Household-level and person-level analysis weights, based on the selection probability at each stage of sampling along with post stratification corrections, were applied to produce nationally representative cohorts [40]. Age and sex standardizations based on WHO’s World Standard Population [51] were carried out to adjust for between-country age and sex differences. The study population in this analysis consisted of adults aged 50-plus from China (*N* = 13,367), Ghana (*N* = 4724), India (*N* = 7150), Mexico (*N* = 2315), Russia (*N* = 3938) and South Africa (*N* = 3840). Computed estimates and odds ratios are reported with 95% confidence intervals and a two-side p-value of 0.05 used as the cut-off point for statistical significance. Associations between key outcomes of edentulism and sociodemographic, chronic conditions, health risk behaviour and other health related variables were evaluated by calculating odds ratios (OR). Unconditional multivariate logistic regression was used for evaluation of the association of explanatory variables for the outcome of edentulism (binary dependent variable). All variables statistically significant at the *p* < 0.05 levels in bivariate analyses were included in the multivariable model. In the analysis, weighted percentages are reported. The reported sample size refers to the sample that was asked the target question. Both the reported 95% confidence intervals and the *p* value are adjusted for the multi-stage stratified cluster sample design of the study. The data were analysed using STATA Version 11 (StataCorp, College Station, TX, USA, 2009).

## 3. Results and Discussion

### 3.1. Sample Characteristics

The overall sample included 34,138 persons 50 years and older from six countries, 52.1% women, 48.4% in the age group 50 to 59 years, 53.8% rural residence, and 29.9% had no and 29.4% had completed nine or more years of formal education. The overall prevalence of edentulism was 11.7%, with India, Mexico, and Russia have higher prevalence rates (16.3%–21.7%) than China, Ghana, and South Africa (3.0%–9.0%) (see Table 1).

**Table 1:** Sample characteristics and prevalence rate of edentulism among older persons in China, Ghana, India, Mexico, Russia and South Africa, SAGE Wave 1.

	Total Sample		Prevalence Rate of Edentulism	
Sociodemographics	N	%	N	95% CI
All	35,334		4124	11.7 (10.7–12.8)
*Country*				
China	13,367	37.8	1416	9.0 (8.0–9.0)
Ghana	4724	13.4	120	3.0 (2.3–3.6)
India	7150	20.2	932	16.3 (14.3–18.4)
Mexico	2315	6.6	524	21.7 (15.5–27.8)
*Country*				
Russian Federation	3938	11.1	739	18.0 (13.7–22.3)
South Africa	3840	10.9	369	8.5 (6.7–10.3)
*Gender*				
Female	19,145	52.1	2467	13.0 (11.8–14.1)
Male	16,180	47.9	1656	10.4 (9.1–11.7)
*Age*				
50–59	14,471	46.4	751	4.9 (4.1–5.7)
60–69	19,969	30.0	1142	10.8 (9.6–11.9)
70–79	7320	18.2	1471	24.2 (21.7–26.8)
80 and over	2574	5.4	760	37.3 (33.2–41.3)
*Educational Level*			1395	17.8 (16.0–19.4)
None	10,582	29.9	772	11.9 (10.3–13.4)
1–4 years	4935	14.0	915	9.7 (7.9–11.4)
5–8 years	7290	20.6	933	7.3 (6.0–8.6)
9 or more years	10,397	29.4		
Missing	2130	6.1		
*Wealth ^1^*				
Lowest	6687	17.0	940	16.9 (14.8–19.0)
Low	6922	18.7	889	13.1 (11.2–15.0)
Medium	6905	19.6	844	12.3 (10.8–13.7)
High	7238	21.8	743	9.4 (7.7–11.0)
Highest	7442	22.6	694	8.6 (7.2–10.0)
Missing	140	0.4		
*Geolocality*				
Rural	17,725	53.8	1954	12.5 (11.3–13.7)
Urban	17,606	46.2	2169	10.7 (8.8–12.5)
Missing	3	0.0		
**Chronic Conditions**				
Angina	3157	8.8	626	16.9 (14.0–19.8)
Arthritis	7079	21.2	1189	14.9 (13.3–16.5)
Asthma	1302	3.5	243	23.0 (17.6–28.5)
Diabetes	2649	6.5	484	13.7 (10.9–16.5)
Hypertension	17,528	52.9	2241	12.7 (11.5–13.9)
**BMI**				
Normal	15,494	14.0	1735	11.1 (9.9–12.2)
Underweight	3637	53.7	541	19.0 (16.2–21.7)
Pre-Obese	8231	24.7	927	9.2 (8.0–10.3)
Obese	4759	7.7	658	11.3 (9.1–13.6)
**Health Risk Behaviour**				
Current daily tobacco use	8210	31.5	987	10.9 (9.4–12.4)
Past daily tobacco use	2899	8.1	437	15.7 (13.7–17.7)
**Health Risk Behaviour**				
Insufficient fruits and vegetables	16,820	38.0	2336	16.1 (14.2–18.0)
**Other Health Related Variables**				
Subjective health status (good/very good)	10,737	31.4	883	8.6 (7.0–10.2)
Poor cognitive functioning	2062	6.4	436	24.3 (20.7–28.0)
*Functional Disability*				
None/Mild	25,553	79.4	2569	9.0 (8.1–10.0)
Moderate	5456	13.1	959	19.9 (17.3–22.5)
Severe/Extreme	1559	3.8	399	32.0 (27.2–36.7)
Missing	2766	3.7		
*Social Cohesion Index*				
Low	9030	39.2	1632	15.4 (13.7–17.1)
Medium	12,375	44.5	1448	10.0 (8.9–11.1)
High	12,065	24.9	1027	10.4 (8.8–12.0)
Missing	1867	1.4		

^1^ Lowest wealth quintile indicates relative economic disadvantage and highest indicates a relative economic advantage.

### 3.2. Association between Socioeconomic and Health Variables and Edentulism

In bivariate analysis, sociodemographic factors (female sex, older age, lower education, lower wealth), chronic conditions (hypertension, angina, arthritis, asthma), BMI underweight, health risk behaviour (former daily tobacco use, inadequate fruits and vegetable consumption) and other health related variables (poor cognitive functioning, poor subjective health status, functional disability and low social cohesion) were found to be associated with edentulism. In multivariate analysis sociodemographic factors (older age, lower education), chronic conditions (arthritis, asthma), health risk behaviour (former daily tobacco use, inadequate fruits and vegetable consumption) and other health related variables (functional disability and low social cohesion) were associated with edentulism (see Table 2).

**Table 2:** Odds ratios for likelihood of edentulism by selected background characteristics and health variables, SAGE Wave 1+.

	Unadjusted Odds Ratio (95% CI)	Adjusted Odds Ratio (95% CI)
**Sociodemographics**		
*Gender*		
Female	1.00	1.00
Male	0.78 (0.68–0.89) ***	0.96 (0.81–1.13)
*Age in Years*		
50–59	1.00	1.00
60–69	2.33 (2.02–2.74) ***	1.98 (1.69–2.31) ***
70–79	6.22 (5.17–7.47) ***	4.78 (4.01–5.71) ***
80 and over	11.55 (9.06–14.72) ***	7.26 (5.74–9.20) ***
*Educational Level*		
None	1.00	1.00
1–4 years	0.63 (0.54–0.73) ***	0.87 (0.72–1.04)
5–8 years	0.50 (0.41–0.62) ***	0.81 (0.67–0.99) *
9 or more years	0.37 (0.30–0.45) ***	0.74 (0.60–0.91) **
*Wealth*		
Lowest	1.00	1.00
Low	0.74 (0.62–0.90) **	0.95 (0.80–1.13)
Medium	0.69 (0.58–0.82) ***	1.03 (0.87–1.23)
High	0.51 (0.41–0.63) ***	0.89 (0.71–1.12)
Highest	0.46 (0.37–0.57) ***	0.85 (0.68–1.06)
**Sociodemographics**		
*Geolocality*		
Rural	1.00	
Urban	0.83 (0.67–1.04)	---
**Chronic Conditions**		
Angina	1.62 (1.30–2.02) ***	1.20 (0.96–1.51)
Arthritis	1.43 (1.24–1.65) **	1.23 (1.06–1.43) **
Asthma	2.35 (1.74–3.18) ***	1.63 (1.14–2.33) **
Diabetes	1.21 (0.96–1.54)	---
Hypertension	1.17 (1.01–1.37) *	1.07 (0.93–1.23)
**BMI**		
Normal	1.00	1.00
Underweight	1.88 (1.56–2.27) ***	1.19 (0.99–1.44)
Pre-Obese	0.81 (0.79–0.94) **	0.91 (0.78–1.06)
Obese	1.03 (0.82–1.29)	0.96 (0.75–1.22)
**Health Risk Behaviour**		
Current daily tobacco use	0.89 (0.78–1.02)	---
Past daily tobacco use	1.45 (1.23–1.72) ***	1.38 (1.09–1.74) **
Insufficient fruits and vegetables	1.97 (1.63–2.37) ***	1.37 (1.10–1.72) ***
**Other Health Related Variables**		
Subjective health status: (very) good	0.63 (0.51–0.76) ***	1.11 (0.91–1.37)
Poor cognitive functioning	2.65 (2.11–3.32) ***	1.16 (0.89–1.51)
*Functional Disability*		
None/Mild	1.00	1.00
Moderate	2.50 (2.12–7.94) ***	1.22 (1.02–1.46) *
Severe/extreme	4.72 (3.72–6.00) ***	1.51 (1.11–2.05) **
*Social Cohesion Index*		
Low	1.00	1.00
Medium	0.61 (0.53–0.71) ***	0.82 (0.70–0.95) *
High	0.64 (0.53–0.78) ***	0.92 (0.76–1.11)

*** *p* < 0.001; ** *p* < 0.01; * *p* < 0.5.

The study found that the prevalence of edentulism in the six study middle income countries seemed lower than in comparable studies in the USA [5,6]. One may need to consider that rates in the US were based on oral exams, while in this study, they were by self-report, which may overestimate the prevalence. There was, however, a wide country variation with high prevalence in Mexico, Russia and India and low prevalence of edentulism in Ghana, South Africa and China. This relatively lower prevalence in Africa is consistent with other studies conducted in other African countries [52,53]. Some authors believe this difference in tooth loss patterns may reflect the fact that African populations tend in general to have much less dental caries [54] due to lower consumption of free sugars compared with industrialised nations such as the US. Thorpe [54], however, argues that levels of edentulism may be artificially low in developing countries due to a shorter life expectancy and thus a much lower percentage of the old and very old population. 

In agreement with other studies [11,12,13,14,15,17], this study found that socioeconomic factors (older age and lower education) were associated with edentulism. Other socioeconomic factors (being female and lower wealth status) were in this study in bivariate analysis associated with edentulism, as found in other studies [6,12,13,17,18]. The role of sex in edentulism has been suggested to be both social and biological. The differences may be related to better dental attendance patterns and dental health behaviour and not just disease occurrence alone. Haikola *et al.* [55] believe that women might appreciate dental, oral and facial appearance more than men and therefore be a motivating factor for attending the clinic. 

Regarding chronic conditions, this study found that having asthma and arthritis were associated with edentulism, as found in some previous studies [20]. In bivariate analysis, also angina and hypertension were found to be associated with edentulism, as also found in Mexico [22] and South Africa [24]. Several other clinical studies have suggested a possible association between rheumatoid arthritis and periodontitis and tooth loss [56]. This association between rheumatoid arthritis and edentulism has been attributed to the shared environmental or genetic risk factors that result in a similar pathobiology. Periodontal disease pathobiology might contribute to the development of rheumatoid arthritis or vice versa [57]. Han *et al.* [58] have attributed the increased tooth loss among asthmatics to the inhalation of corticosteroids, resulting in a reduced bone mineral density.

Hypertension and poor cognitive function and being underweight showed no association with edentulism though there was an association in the bivariate analysis diabetes, however, showed no association with edentulism though other studies have reported an association. Dietary factors have been implicated in the etiology of cognitive decline and dementia [59], diabetes, and cardiovascular disease. And dental health is an important determinant of nutritional status, body mass index (BMI), and general health in older people [60,61]. Some studies have observed that edentulism causes individuals to alter their diet, resorting to a diet that is low in fiber and high in saturated fat [62], putting them at a higher risk of being obese and developing these noncommunicable disease. Lee *et al.* [63] demonstrated that edentulism was associated with a weight gain of >5% in one year. In this study however, we found the reverse with edentulous individuals being more underweight rather than overweight. Thus, though hypertension, angina, and diabetes have been associated with tooth loss in other studies mainly in developing countries it wasn’t so in these middle income countries and may be attributed to dietary factors. The diet and dietary habits of edentulous individuals in these middle income countries may not be putting them at more risk of these heart diseases, diabetes or dementia. Furthermore the role of socioeconomic factors influencing this association cannot be ignored, with dietary habits among edentulous individuals may thus have to be assessed further as their diet may not be one of high risk for cardiovascular disease as compared to other studies. Alternatively, this study utilized a self report of diagnosed chronic conditions, this finding could be because there is a high prevalence of undiagnosed hypertension and diabetes in most of these middle income countries accounting for this finding.

Among health risk behaviours former smoking and inadequate fruits and vegetable consumption, as found in previous studies [15,26,29,30], were associated with edentulism. In this study only former smoking and not current smoking was associated with edentulism. It is possible that former smokers were heavier smokers than current smokers increasing the effect on tooth loss, as also found in some other studies [15,26]. In India, Reddy *et al.* [64] found that a mixed diet population had a higher level of edentulism compared with vegetarians. Edentulous individuals also were observed to be consuming inadequate amounts of fruits and vegetables necessary to provide adequate nutritional intake to maintain good health. Josipura *et al.* [65] noted a similar observation when they demonstrated that, compared to dentate individuals, edentulous respondents consumed fewer vegetables, less fibre, and less carotene intake, while consuming more cholesterol and saturated fats. This finding may thus predispose these edentulous individuals with chronic diseases. However, it cannot be assumed that low intake of fruits and vegetables in the edentulous is a result of the limited masticatory function. A high intake of fruits and vegetables has been shown to be associated with lower risk of periodontal disease, a major cause of tooth loss [66], which may mean that a low intake of fruits and vegetables may be an indirect cause edentulism. Dietary sugars are the most important factor in the development of dental caries, a major cause of tooth loss. In this study, it was not possible to explore whether intake of free sugars was associated with edentulism as the limited dietary data did not include information on sugar intake. 

Consistent with another study [31], functional disability was in this study found to be associated with edentulism. It has been demonstrated that having fewer than 10 contacting pairs of upper and lower teeth yield impaired masticatory efficiency and are likely to result in a reduction in reported masticatory ability [67]. Unlike in some other studies [32,33], this study did not find poor cognitive functioning to be associated with edentulism. However, this study observed a significant association with functional disability, similar to a study by Avlund *et al.* [68]. Functional disability may pose a challenge with carrying out proper oral hygiene practices and with access to receiving dental care, thus predisposing them to dental disease and tooth loss. 

Individuals belonging to social networks are more likely to follow health-enhancing behaviours and to have higher self-esteem and, hence, have better health [34]. In adults, social support has been observed to be a determinant of oral health-related quality of life [37] and psychosocial factors, such as loneliness and social isolation, were associated with onset of periodontal disease [38]. Social networks are also associated with better self-rated oral health, and social capital had beneficial effects on the number of teeth [39]. Poorer social support was found to be associated with having fewer functioning teeth, worse dental behaviours, and more periodontal attachment loss [68]. 

The ageing population is increasing globally—one in nine persons worldwide are aged 60 years and over and it is predicted that by 2050 this will increase to one in five people in developing countries [69]. This, coupled with the observed levels of edentulism in middle income countries suggests that edentulism and its impact on health and wellbeing will remain a significant health challenge to these nations.

### 3.3. Study Limitations

This study had several limitations. Firstly, the questionnaire was interviewer-administered, it is possible that some study participants may have mis-reported either intentionally or inadvertently on any of the questions asked. Intentional miss-reporting was probably minimised by the fact that study participants completed the questionnaires anonymously. Edentulism was only assessed with a single question. Oral examinations assessing the number of teeth and saliva flow [35] are important in future studies. In addition, some other measures relied on a single question, such as subjective health and cognitive functioning, which have its limitations. Furthermore, this study was based on data collected in a cross sectional survey. We cannot, therefore, ascribe causality to any of the associated factors in the study. Finally, the analysis was limited to the variables included in SAGE, and other factors such as undernutrition (e.g., inadequate percentage body fat), inadequate oral hygiene and sugar intake [29,70] found significant in previous studies should be included in future research.

## 4. Conclusions

The national estimates and identified risk factors of edentulism among older adults across the six countries can help policy makers and public-health researchers to understand the importance of edentulism and it overall impact on the health of elderly. This study can help to advance the need for health programmes focusing on elderly that are also inclusive of oral health promotion and prevention.
